# On-Site Calibration Method for Line-Structured Light Sensor-Based Railway Wheel Size Measurement System

**DOI:** 10.3390/s21206717

**Published:** 2021-10-09

**Authors:** Yunfeng Ran, Qixin He, Qibo Feng, Jianying Cui

**Affiliations:** 1 Key Laboratory of Luminescence and Optical Information, Ministry of Education, Beijing Jiaotong University, Beijing 100044, China; 16118442@bjtu.edu.cn (Y.R.); heqixin@bjtu.edu.cn (Q.H.); jycui1@bjtu.edu.cn (J.C.); 2 Dongguan Nannar Electronic Technology Company Ltd., Dongguan 523050, China

**Keywords:** structured light, on-site calibration, wheel size measurement

## Abstract

Line-structured light has been widely used in the field of railway measurement, owing to its high capability of anti-interference, fast scanning speed and high accuracy. Traditional calibration methods of line-structured light sensors have the disadvantages of long calibration time and complicated calibration process, which is not suitable for railway field application. In this paper, a fast calibration method based on a self-developed calibration device was proposed. Compared with traditional methods, the calibration process is simplified and the calibration time is greatly shortened. This method does not need to extract light strips; thus, the influence of ambient light on the measurement is reduced. In addition, the calibration error resulting from the misalignment was corrected by epipolar constraint, and the calibration accuracy was improved. Calibration experiments in laboratory and field tests were conducted to verify the effectiveness of this method, and the results showed that the proposed method can achieve a better calibration accuracy compared to a traditional calibration method based on Zhang’s method.

## 1. Introduction

In recent years, line-structured light vision sensors have been widely used in dynamic railway wheel size measurement systems [1,2,3,4,5,6]. For example, a high-accuracy line-structured light sensor-based wheel size measurement system was introduced in our previous work [7]. A line-structured light vision sensor is generally composed of a camera and a line laser projector. In the application of railway wheel size measurement, owing to the restriction of view angle, it is necessary to combine at least two sensors whose laser planes are coincident to obtain a whole wheel tread profile. Calibration is one of the crucial phases to realize the wheel parameters reconstruction by the acquired 2D laser strips, which is vital to improving the accuracy of the system. Generally, the calibration parameters of a line-structured light vision sensor consist of the intrinsic parameters of the camera and the light plane parameters. The calibration of camera intrinsic parameters has been wildly studied [8,9,10,11,12,13]; thus, this paper mainly focuses on the calibration of light plane parameters.

Xie [14] used a planar target with grid lines to calibrate the intrinsic and light plane parameters simultaneously. During the calibrating process, the intersection points between the grid lines of the planar target and laser lines are extracted as calibration points. Liu [15] adopted a ball target with high roundness to calibrate the laser plane. First, the spatial cone equation and the sphere equation of the ball target are solved. Then, the solution of the light plane equation is obtained by nonlinear optimization. Huynh [16] created a V-shape 3D target for laser plane calibration. The sensor is mounted on an AGV to scan the target in calibration. The position of sensor related to the world coordinate frame is known. According to cross-ratio invariability, the laser plane equation can be solved by combining the 3D coordinates of points of the target. Xu [17] employed a flat board target with four balls. The orientation of the board plane is first solved by the four balls, and then the intersection line between the board plane and the laser plane is obtained. The laser plane equation is fitted by these intersection lines. Xie [18] similarly utilized a flat board target with squares pattern and solved the orientation of the board plane by the corner points. Differently, the angle of the board plane and laser plane is computed by an additional raised block on the board target. Wei [19] proposed a method based on a 1D target. The feature points of the target are calculated in the camera coordinate frame using the known distance constraint of target pattern. Then, the nonlinear optimization method is used to solve the plane feature points and the light plane equation can be fitted.

The above methods have achieved good results in laboratory environment, but it is not suitable for railway field application. The calibration of an on-site railway wheel size measurement system has the following characteristics: (1) the available calibration time is limited to the busy railway operations; (2) the calibration accuracy is influence by the strong natural light on the outdoor environment; (3) the depth of field of vision sensors is short, making it difficult to shoot calibration markers placed on different locations. To achieve fast, high-accuracy on-site calibration of a wheel size measurement system, a new calibration method is demonstrated in this paper, and the above issues in field calibration are solved. This method shortens the calibration time, overcomes the problem caused by short depth of field, and does not need to extract laser lines, avoiding the influence of natural light. In order to realize the calibration method, a specific calibration device was developed. In calibration, the calibration device is mounted on the rail, and the calibration board plane is manually adjusted to coincide with the light plane. Then, the pixel coordinates of corner points are abstracted, and the fitting equations of image coordinates and lase plane coordinates are calculated. Finally, a calibration revising method based on epipolar constraint is employed to reduce the calibration error and improve the data fusion effect.

In Section 2, the calibration device and the principle of the proposed calibration method are introduced. In Section 3, a corner extraction method for calculating the calibration parameters is proposed, and the calibration errors caused by the extraction process are analyzed. Then, the revising method of calibration parameters is described in Section 4. The epipolar constraint is used to find matching points, laying a foundation for establishing constraint equations in calibration parameters calculation. In Section 5, the results of the physical experiment are presented, and the calibration accuracy is evaluated by comparison. Finally, conclusions are drawn in Section 6.

## 2. Calibration Principle

**Figure 1:**
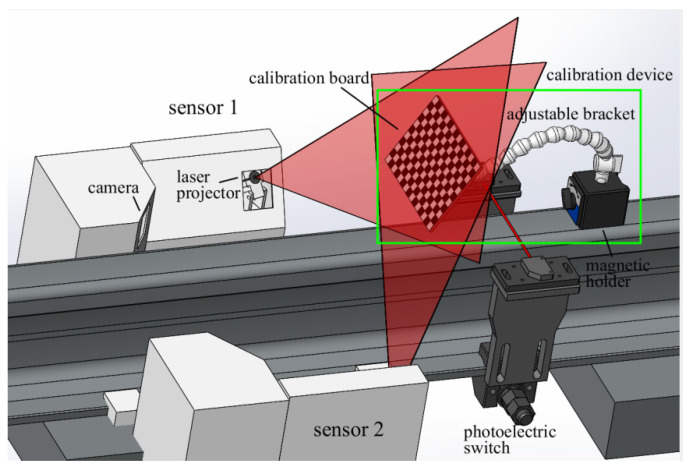
Schematic of the calibration process.

Figure 1 illustrates the setup of calibration by our method. Sensor 1 and sensor 2 are both line-structured light vision sensors; the two laser planes are carefully adjusted to be coincident for measuring the wheel tread size together. This on-site wheel tread size measurement system is demonstrated in our previous work [7]. The system can reach 0.11 mm theoretical measurement accuracy at the designed 300 mm work distance. The maximum frame rate of the camera is 20 fps, which is enough to meet the requirement of dynamic measurement under 48 km/h. When the train passes, the photoelectric switch triggers the camera to grab images. Then, the image is transmitted to computers and processed to extract laser stripes. Here, the purpose of the calibration is to establish a criterion of transforming the laser stripes to three-dimensional reconstruction profiles.

The calibration device is composed of a magnetic holder, a calibration board and an adjustable bracket composed of multiple cardan joints. The adjustable bracket allows the calibration board to move and rotate in space and be fixed when the adjustment is finished. During calibration, the magnetic holder is fixed on the rail and the plane of the calibration plate is placed to coincide with the light plane by adjusting the adjustable bracket. In experiment, the laser light covering the whole board can be seen as a sign that the coincidence degree of two planes meets the requirements. The calibration method only needs to take one shot of the calibration plate, and then the image of the calibration pattern is obtained by the camera, and the corner points in the calibration pattern are extracted.

**Figure 2:**
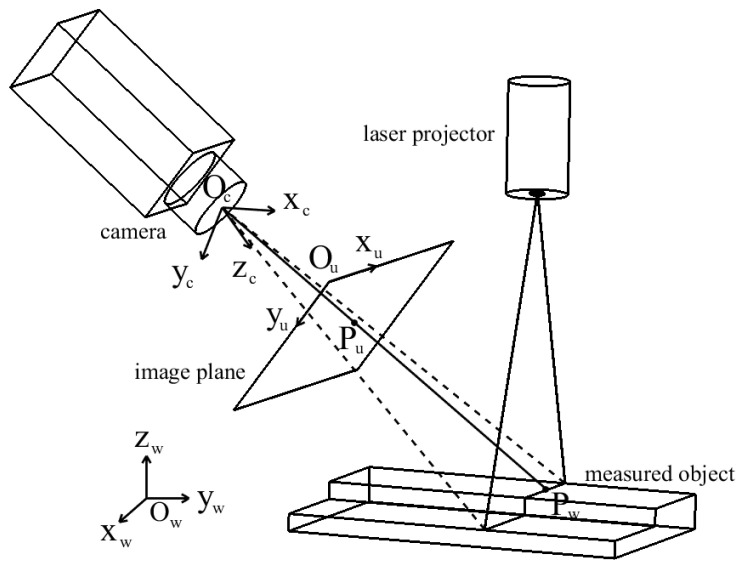
Schematic of the line-structured light vision sensor.

The schematic of the line-structured light vision sensor is exhibited in Figure 2. In this figure, *O_w_x_w_y_w_z_w_* represents the world coordinate frame (WCF), *O_c_x_c_y_c_z_c_* indicates the camera coordinate frame (CCF), and *O_u_x_u_y_u_* refers to the image coordinate frame (ICF). Assume that an arbitrary point *P_w_* = [*x_w_*,*y_w_*,*z_w_*,1]*^T^* in WCF has a projection *P_u_* = [*x_u_*,*y_u_*,1]*^T^* in ICF. According to the camera imaging model and disregarding distortion, it can be expressed as:

(1) $$sP_{u}=A[\begin{array}{cc} R & t \end{array}]P_{w}$$

where *s* denotes the size factor, *A* is the camera’s intrinsic parameters matrix, *R* and *t* refer to the rotation matrix and translation vector from WCF to CCF, respectively. The equation can realize the transformation from WCF to ICF. In order to reconstruct a 3D profile of the measured object, the equation is combined with the light plane equation to transform a coordinate from ICF to WCF, that is:

(2) $$\{\begin{array}{c} sP_{u}=A[\begin{array}{cc} R & t]P_{w} \end{array}\\ ax_{w}+by_{w}+cz_{w}+d=0 \end{array}$$

When line-structured light vision sensors are applied to measure the object size, the stipulation of WCF is irrelevant. The *O_w_x_w_y_w_* plane of WCF can be set as the light plane π. Thus, when reconstructing 3D profile, *z_w_* = 0. The functional relationship between (*x_w_*, *y_w_*) and (*x_u_*, *y_u_*) can be simply expressed as *x_w_*~(*x_u_*, *y_u_*), *y_w_*~(*x_u_*, *y_u_*), which can be obtained by fitting. In this paper, the selected polynomial basis is shown as follows:

(3) $$\{\begin{array}{c} x_{w}=\sum_{i}^{m}\sum_{j=0}^{i}c_{ij}x_{u}^{i}y_{u}^{i-j}\\ y_{w}=\sum_{i}^{m}\sum_{j=0}^{i}q_{ij}x_{u}^{i}y_{u}^{i-j} \end{array}$$

where *m* indicates the highest power of the polynomial. In our proposed calibration method, the calibration board plane is adjusted to coincide with the light plane. Therefore, a set of (*x_w,i_*,*y_w,i_*) and (*x_u,i_*, *y_u,i_*) used for fitting can be obtained by the manufacturing dimensions of the calibration board and the extraction of corner points. The coefficients of polynomials are acquired based on the least-square principle [20]:

(4) $$t={\left(V^{T}V\right)}^{-1}V^{T}L$$

where *V* represents the Vandermonde matrix.

(5) $$V_{ij}=x_{u,i}^{k_{1}}y_{u,i}^{k_{2}},k=\max(k,k\in j-\sum_{m=0}^{k}m>0),k_{1}=j-\sum_{m=0}^{k}m-1,k_{2}=k-k_{1}$$

where *L* represents the vector [*x_w,i_*]*^T^* and [*y_w,i_*]*^T^*, and t denotes the vector of the coefficients of polynomials.

## 3. Corner Extraction and Influence of Image Noise

**Figure 3:**
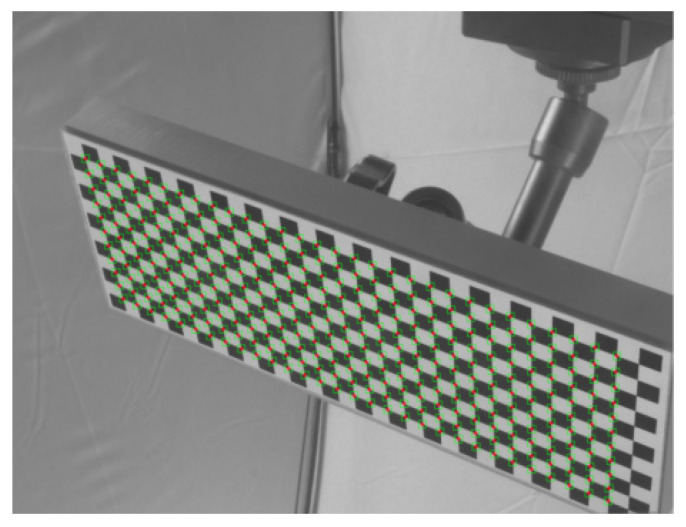
Corner points detected by Harris corner detection algorithm.

The Harris corner detection algorithm is widely used to detect corner points on the image. The basic idea of the algorithm is to use a fixed window to slide on the image and compare the change of gray values in the window before and after sliding. If there is a large gray change in any direction sliding, there will be a corner point in the window. Here, the Harris corner detection algorithm is employed to obtain the preliminary rough image coordinates (*x*_*u*0_, *y*_*u*0_) of corner points, as presented in Figure 3. The precise image coordinates of the corner points can be obtained by the following iterative process [21]:

(6) $$\left(\begin{array}{c} x_{u,i+1}\\ y_{u,i+1} \end{array}\right)=\frac{\left(\begin{array}{cc} \sum_{w}g_{yy} & -\sum_{w}g_{xy}\\ -\sum_{w}g_{xy} & \sum_{w}g_{xx} \end{array}\right)\left(\begin{array}{c} \sum_{w}g_{xx}x_{u}+g_{xy}y_{u}\\ \sum_{w}g_{xx}x_{u}+g_{xy}y_{u} \end{array}\right)}{\left|\begin{array}{cc} \sum_{w}g_{yy} & -\sum_{w}g_{xy}\\ -\sum_{w}g_{xy} & \sum_{w}g_{xx} \end{array}\right|}$$

(7) $$g_{xx}(x_{u},y_{u})=g_{x}^{2}\omega (x_{u},y_{u}),g_{xy}(x_{u},y_{u})=g_{x}g_{y}\omega (x_{u},y_{u}),g_{yy}(x_{u},y_{u})=g_{v}^{2}\omega (x_{u},y_{u})$$

where *w* represents the detection window with the center of (*x_u,i_*,*y_u,i_*), *g_x_*(*x_u_*,*y_u_*) and *g_x_*(*x_u_*,*y_u_*) indicate gray gradients along *x_u_* and *y_u_* direction, respectively, and ω(*x_u_*,*y_u_*) denotes the two-dimensional Gaussian distribution function:

(8) $$\omega (x_{u},y_{u})=e^{\frac{{\left(x_{u}-x_{u,i}\right)}^{2}+{\left(y_{u}-y_{u,i}\right)}^{2}}{2\sigma^{2}}}$$

**Figure 4:**
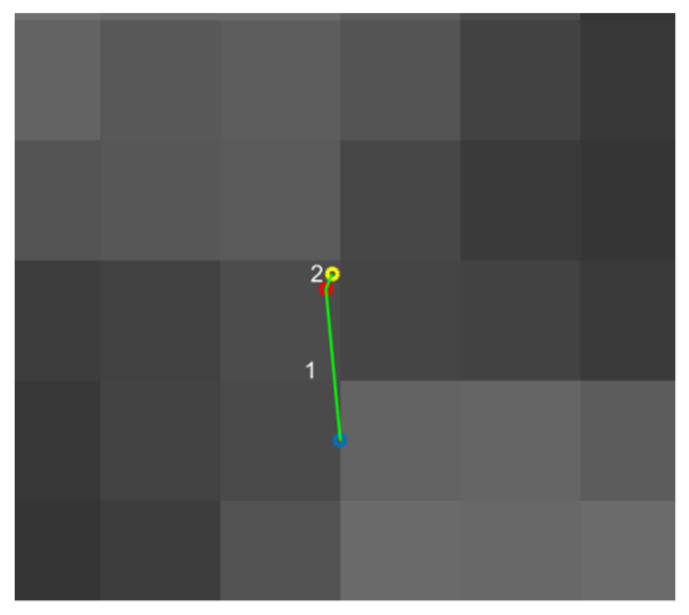
The iterative process of extracting corner points starting from the result of the Harris detection algorithm (blue point).

In most cases, the iterative accuracy of 0.005 pixels can be achieved after two or three iterations. The iterative process is shown in Figure 4.

**Figure 5:**
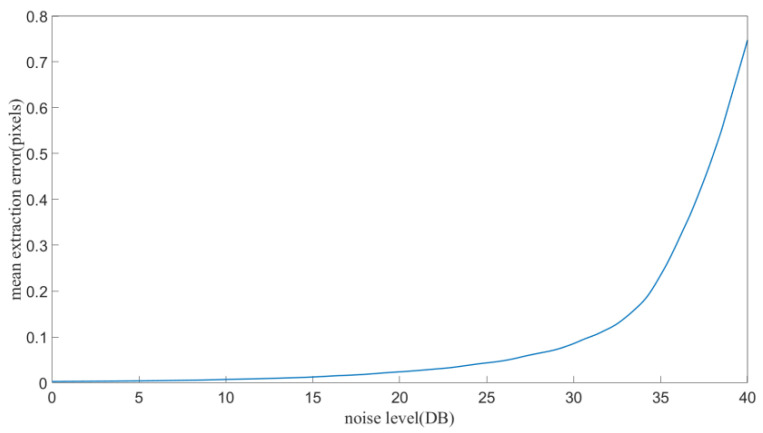
The mean extraction error of corner points at different noise levels.

A standard calibration plate pattern image was generated by computer program to estimate the accuracy of the corner extraction. Gaussian noise was added to the standard image with a different noise level varies from 0 to 40 DB at an interval of 0.1 DB. For each noise level, 50 experiments were conducted, and the extraction error was computed and shown in Figure 5. It can be seen that the extraction accuracy increases as the noise decreases.

**Figure 6:**
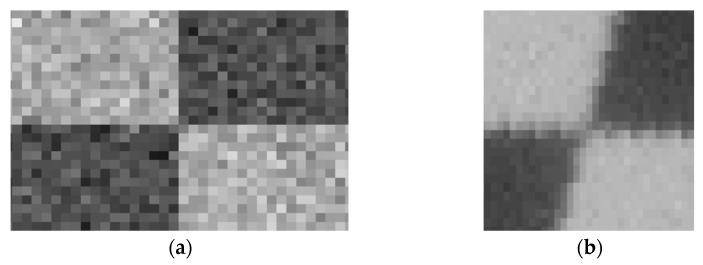
Comparison between the simulation image and the real image (local region nearby the corner point). (**a**) Simulation image (at 35 DB noise level); (**b**) real image.

**Figure 7:**
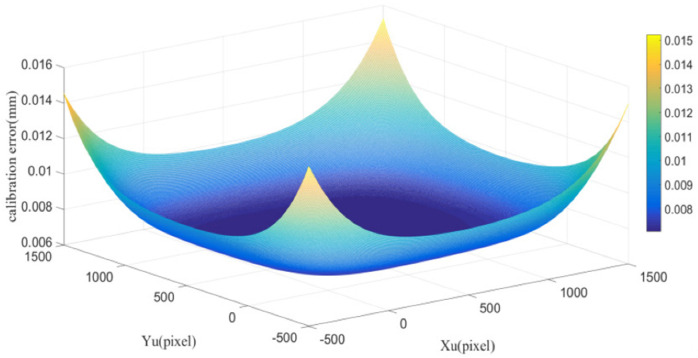
Calibration error caused by the image noise (35 DB).

For a real calibration image, there is an inevitable gradual change at the black-and-white boundary due to manufacturing reasons. Therefore, the noise level at corner areas is relatively higher than that at homogenous areas. For estimating the extraction accuracy of corner points, small areas around corner points were intercepted from the simulation calibration image and the real calibration image as shown in Figure 6. According to previous studies of image noise estimation [22,23,24], the noise level of the acquired real calibration image at corner areas is equal to the simulation calibration image with 35 DB Gaussian noise. Therefore, the extraction accuracy of corner points for our setup is about 0.2 pixels. The calibration error caused by the image noise is simulated as shown in Figure 7. In the simulation, the calibration plate, the square of the calibration plate, the image size was set to 100 × 100 mm, 5 × 5 mm and 1000 × 1000 pixel, respectively. The extraction error of 0.2 pixels was set to random different directions, and then the mean calibration error was calculated.

In this experiment, the calibration error caused by the corner extraction error is small in the plate area (Xu and Yu direction in 0–1000 pixels range) and increases rapidly out of this area. Therefore, the calibration plate should include the whole measurement range of the sensor to obtain a higher calibration accuracy.

**Figure 8:**
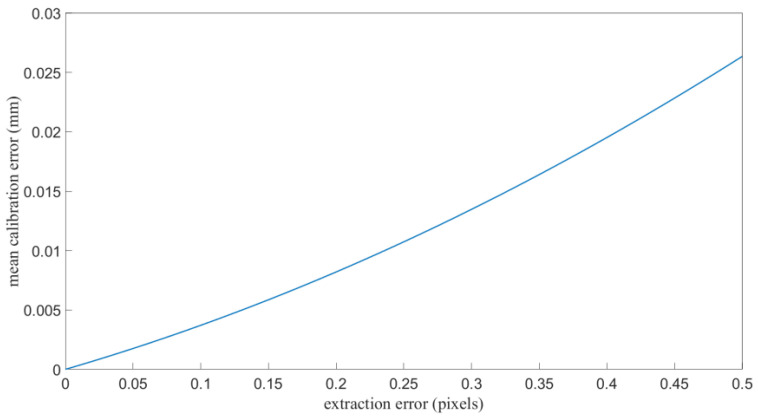
Mean calibration error in the plate area with different extraction error.

Consider that the image noise level varies with camera parameters, shutter speed and amount of ambient light, the extraction error also varies in different application environment. Extra simulation experiments were conducted, and the average calibration error in the plate area caused by different extraction error were calculated. As shown in Figure 8, when the extraction error is up to 0.5 pixels, which only happens at extreme image noise level, the average calibration error in the plate area is 0.025 mm. At this case, the calibration setup should be adjusted to obtain a lower image noise level.

## 4. Calibration Revising Based on Epipolar Constraint

When the measured object is a train wheel, it has to combine at least two line-structured vision sensors with co-planar laser planes because of the restriction of view angle. In practice, it is difficult to adjust the laser planes to be completely co-planar, and there is always a small angle between them. Thus, the calibration planes cannot be adjusted to be co-planar with both laser planes, leading to a certain calibration error. This calibration error leads to misalignment of reconstructed sections, which will bring problems to further calculation.

**Figure 9:**
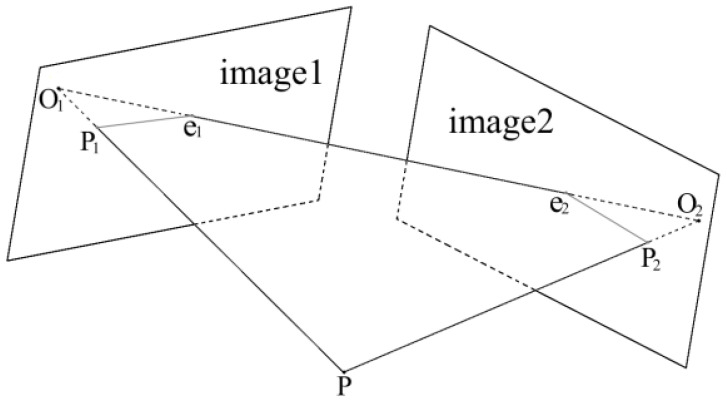
Geometric model of the epipolar constraint.

In order to decrease these calibration errors, an epipolar constraint-based revising method was employed. First, the matching points of two acquired images are found by the epipolar constraint. Then, additional constraint equations based on matching points are added to the calculation of calibration parameters. The constraint of image point and camera optical center is formed in the projection model when the same point is projected onto two images with different viewing angles. As shown in Figure 9, the line O_1_O_2_ connecting the optical centers of the two cameras is called baseline, the intersection points of the baseline and image planes (e_1_ and e_2_) are called base points, and the plane O_1_O_2_P is called polar plane. If the projection point of P on image1 and image2 is denoted as P_1_ and P_2_, the projection point P_2_ must be on the intersection line e_2_P_2_ of polar plane O_1_O_2_P and image2 plane. The intersection line e_2_P_2_ is called the epipolar line.

The epipolar constraint can be expressed as:

(9) $$p_{k}^{T}{Fp}_{k}^{\prime }=0\;(k=1,2,\cdots ,n)$$

where $p_{i}=(x_{u,k},y_{u,k},1)$ and $p_{i}^{\prime }=(x_{u,i},y_{u,i},1)$ indicate the projection points on image1 and image2 of the same point. The basic matrix *F* can be solved based on the least-square principle and the corner points extracted in the calibration process. For a point $p^{\prime }$ on image2, the epipolar line *L*_1_ of camera1 can be expressed as:

(10) $$L_{1}=Fp^{\prime }$$

Regarding a certain object captured by the line-structured light vision sensor, the corresponding point $p_{k}$ on image1 of the point $p_{k}^{\prime }$ on image2 must be the intersectionpoint of the laser stripe on image1 and the epipolar line *L*1, which is useful to find matching points. In experiment, the captured object is the train wheel. Based on these matchingpoints, constraint equations are introduced into Equation (3), which can be expressed as:

(11) $$\{\begin{array}{c} \sum_{i}^{m}\sum_{j=0}^{i}c_{ij}x_{u,k}^{i}y_{u,k}^{i-j}=\sum_{i}^{m}\sum_{j=0}^{i}c_{ij}^{\prime }x_{u,k}^{i}y_{u,k}^{i-j}\\ \sum_{i}^{m}\sum_{j=0}^{i}q_{ij}x_{u,k}^{i}y_{u,k}^{i-j}=\sum_{i}^{m}\sum_{j=0}^{i}q_{ij}^{\prime }x_{u,k}^{i}y_{u,k}^{i-j} \end{array}$$

**Figure 10:**
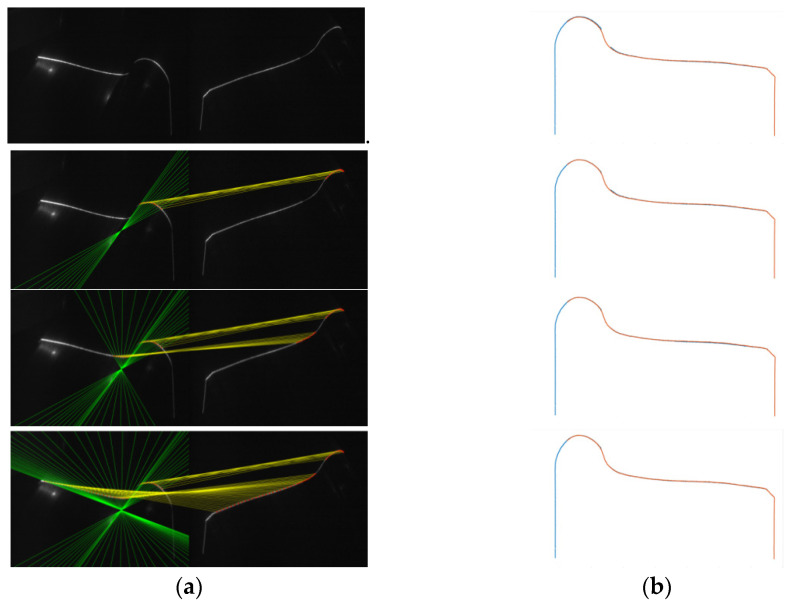
Calibration revising results based on different amounts of matching points. (**a**) Matching points of two images (matching points are connected by yellow lines and green lines are epipolar lines). (**b**) Reconstructed profiles after calibration parameters being revised according to the matching points.

The matching points were found according to the epipolar constraint and exhibited in Figure 10a together with the corresponding epipolar lines. The results of the calibration revising process are presented in Figure 10b. Since the matching points are introduced to calculate calibration parameters, the corresponding parts of the reconstructed profiles become coincident. After choosing enough and proper matching points, the reconstructed profiles of the two sensors are coincident, making it more accurate for further calculation.

## 5. Physical Experiment

The line-structured light vision sensor-based wheel size measurement system was introduced in our previous paper [7]. In the experiment, the wheel size measurement system was calibrated by the proposed calibration method and a comparison method.

**Figure 11:**
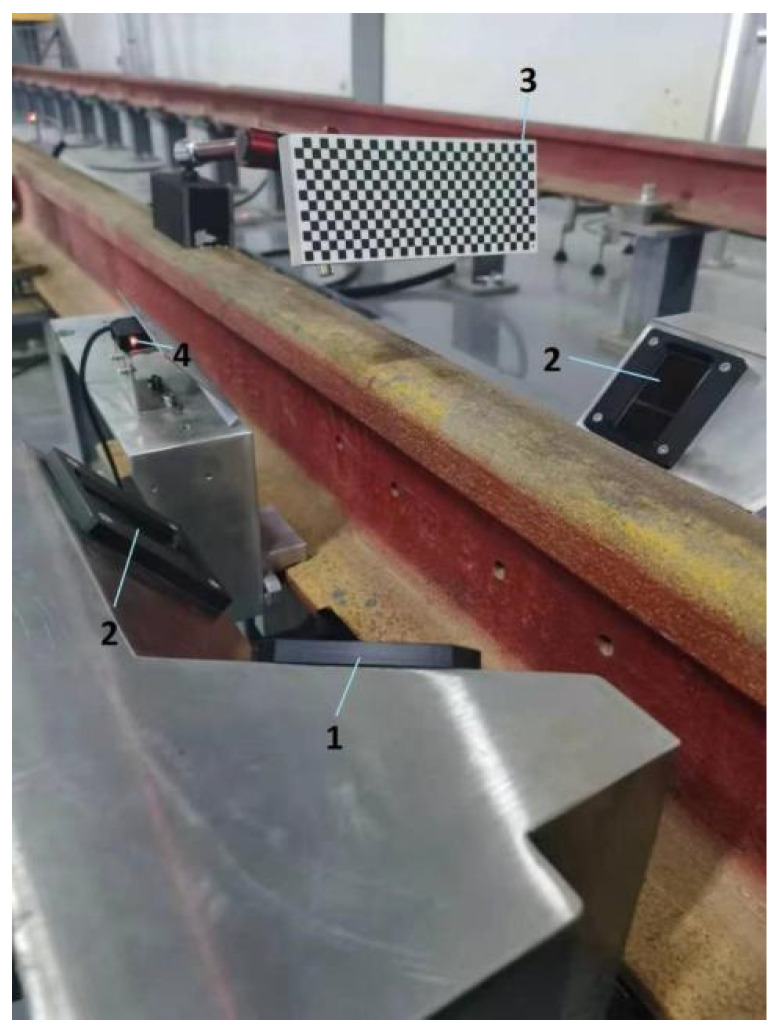
The arrangement of calibration. (1) Camera window; (2) laser window; (3) calibration board; (4) photoelectric switch.

The calibration arrangement is shown in Figure 11. The two laser planes are carefully adjusted to make them as coplanar as possible. The pixel size of the camera is 4.4 × 4.4 μm, the image resolution is 1236 × 1626 pixels, and the lens focal length is 16 mm. The cameras have a FOV of 180 × 135 mm at a working distance of 300 mm. The size of the calibration plate is 160 × 60 mm, the square size is 5 × 5 mm, and the manufacturing precision is 0.003 mm. Furthermore, our proposed method is compared with another method based on Zhang’s method [25] to verify its efficiency.

In the first experiment, the line-structured light vision sensor is calibrated by our proposed method. The plane of the calibration plate is adjusted to coincide with the light plane, and the calibration pattern is adjusted to contain the measuring area, as to reduce the calibration error caused by the corner extraction error. The calibration polynomial coefficients before and after epipolar constraint revising are displayed in Table 1.

**Figure 12:**
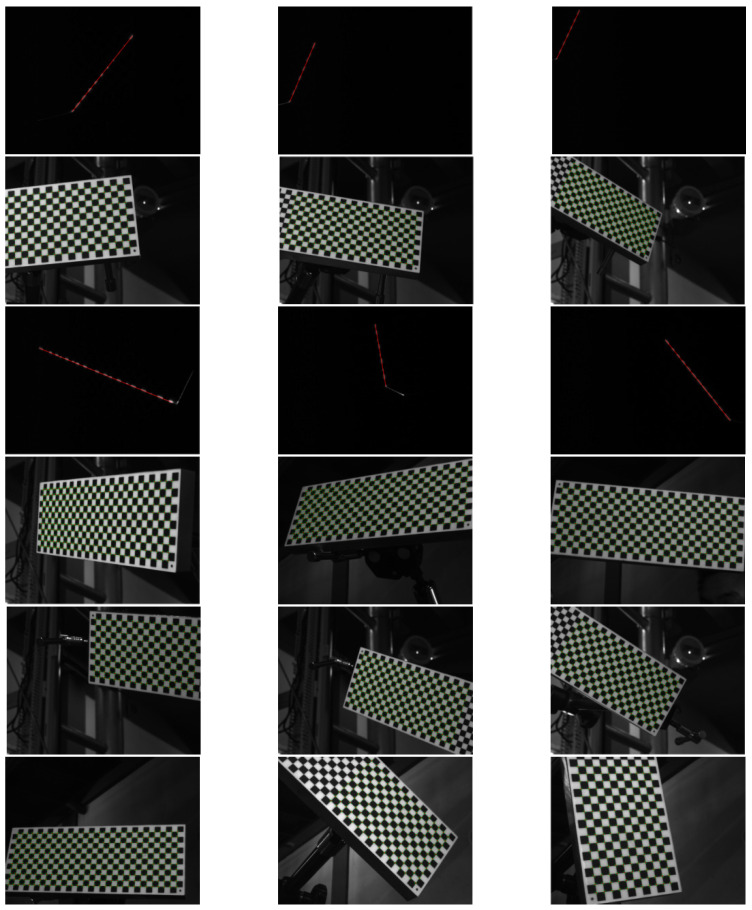
Images used for calibration in the second experiment.

**Figure 13:**
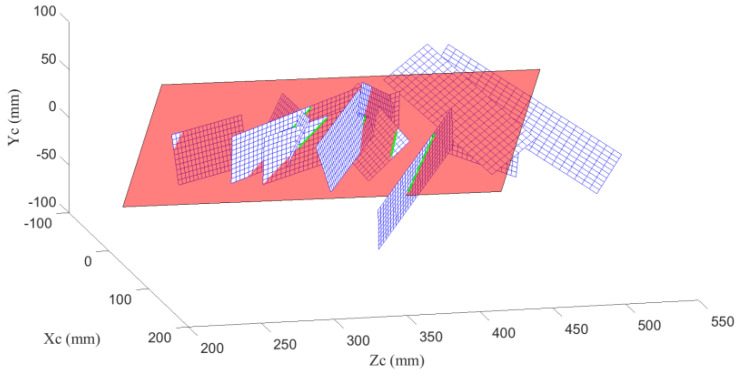
The extrinsic parameters and the fitting laser plane (red) in the second experiment.

In the second experiment, the calibration plate is placed in different locations and directions 12 times. The cameras grab two images each time: one shot with natural light and the other with laser light. The intrinsic parameters of the camera are solved by Zhang’s method using the images in natural light, the extrinsic parameters (representing the locations and directions of the calibration plate) are also calculated. The laser stripes on images are extracted and the coordinates of the laser stripes in CCF can be solved according to the extrinsic parameters. Then, the laser plane equation in CCF can be obtained by fitting these coordinates as a plane, and the calibration is completed. The images used for the calibration are displayed in Figure 12. The extrinsic parameters and the fitted laser plane are illustrated in Figure 13. The calibration results are exhibited in Table 2.

Furthermore, a planar target with grid lines in a horizontal direction is adopted to compare the two calibration methods. The target is placed in the measuring region of the line-structured light vision sensor three times with different orientations. The coordinates of intersection points between the laser stripe and the grid lines are extracted from images. These coordinates are transformed to CCF or WCF by the two calibration methods separately. Then, the distance of intersection points and the angle between the laser stripe and the grid lines are calculated. Additionally, the widths of grid lines on the planar target are calculated as *w_m_*. The fabricated widths of grid lines with a precision of 0.01 mm are regarded as ideal widths *w_i_*. In this experiment, three pairs of intersection points on the planar target are selected each time. The comparison of *w_m_* and *w_i_* are displayed in Table 3.

The calibration accuracy of the proposed method before epipolar constraint revising is approximately 0.052 and 0.057 mm under a measurement range of 150 × 50 mm on camera 1 and camera 2, respectively. After epipolar constraint revising, the calibration accuracy of the proposed method is improved to 0.034 and 0.033 mm. Moreover, the calibration accuracy of the compared method in experiment 2 is 0.048 mm. As revealed by checking the used images, the calibration accuracy of the compared method is relatively low versus that of the proposed method due to the image blur caused by the short depth of field.

**Figure 14:**
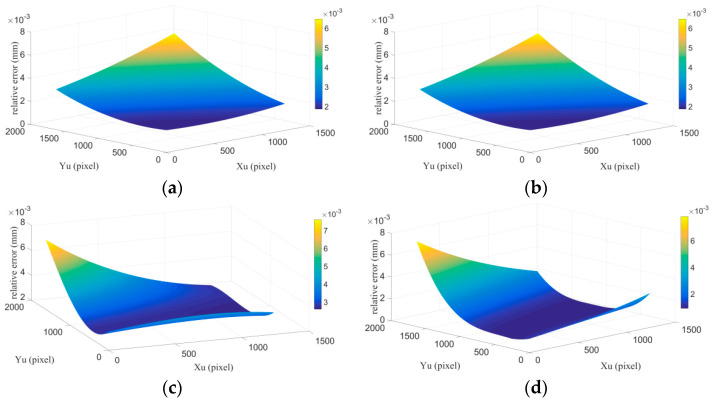
The repeatability experiment results. (**a**–**d**) represent the relative errors of the four experiments compared with experiment 1 at different pixel coordinates.

To verify the reproducibility of our method, the calibration device was removed and then reinstalled four times. Each time, the calibration parameters were recalculated and revised by epipolar constrain. The relative error compared to the experiment 1 at different pixel coordinates are shown in Figure 14. The maximal relative error of the four measurements is 0.008 mm, that is, the repeatability error is within 0.016 mm.

## 6. Conclusions

The coordinates of the calibration plate can represent the coordinates of the laser plane when the calibration plate plane coincides with the laser plane. Based on this feature, a fast line-structured light vision sensor calibration method is proposed in this paper. In addition, the calibration error is revised based on the epipolar constraint to improve the accuracy of calibration. The basic principle and the implementation of the proposed method are described in detail. Then, the proposed method is validated by experiments.

The advantages of the proposed method are described as follows. (1) The proposed method is easy to perform and time-saving, suitable for line-structured light vision sensors used on special environment which is hard to maintain such as the railway site. (2) The proposed method does not need to extract laser lines on images and can adapt to the outdoor environment under strong natural light. (3) The proposed method can avoid the image blur caused by the depth of field as one image on the working distance is enough to accomplish the calibration process.

## Figures and Tables

**Table 1:** Calibration polynomial coefficients of the proposed method.

	Camera 1				Camera 2			
	Xw Direction		Yw Direction		Xw Direction		Yw Direction	
Term	Unrevised	Revised	Unrevised	Revised	Unrevised	Revised	Unrevised	Revised
1	156.5944	156.5728	91.3905	91.3099	166.5998	166.2415	128.0307	127.7353
x	−0.1439	−0.1438	0.0406	0.0406	−0.0901	−0.0897	−0.0227	−0.0224
y	0.0407	0.0404	−0.1276	−0.1275	−0.0227	−0.0213	−0.1049	−0.1037
xy	−5.1677 × 10^−5^	−5.1809 × 10^−5^	4.5263 × 10^−5^	4.5539 × 10^−5^	1.0317 × 10^−5^	9.5430 × 10^−6^	1.5862 × 10^−5^	1.5247 × 10^−5^
x²	3.8892 × 10^−5^	3.8857 × 10^−5^	−1.0673 × 10^−5^	−1.0718 × 10^−5^	4.0923 × 10^−5^	4.000 × 10^−5^	8.8862 × 10^−5^	8.8043 × 10^−5^
y²	1.1971 × 10^−5^	1.2321 × 10^−5^	−3.5261 × 10^−5^	−3.5646 × 10^−5^	1.005 × 10^−5^	8.6387 × 10^−6^	1.1518 × 10^−5^	1.0409 × 10^−5^
xy²	−1.6359 × 10^−8^	−1.6387 × 10^−9^	2.9713 × 10^−8^	2.9321 × 10^−8^	−1.8560 × 10^−8^	−1.8135 × 10^−8^	−2.6934 × 10^−8^	−2.6594 × 10^−8^
x²y	2.0933 × 10^−8^	2.1044 × 10^−8^	−2.0630 × 10^−8^	−2.0421 × 10^−8^	−2.272 × 10^−8^	−2.2595 × 10^−8^	−1.6565 × 10^−8^	−1.6470 × 10^−8^
x³	−9.2042 × 10^−9^	−9.2251 × 10^−9^	4.4243 × 10^−9^	4.3744 × 10^−9^	−9.4866 × 10^−9^	−9.4757 × 10^−9^	−2.7881 × 10^−9^	−2.7759 × 10^−9^
y³	3.0349 × 10^−9^	2.8942 × 10^−9^	−1.5195 × 10^−8^	−1.4898 × 10^−8^	−4.1568 × 10^−9^	−3.6499 × 10^−9^	−1.6091 × 10^−8^	−1.5713 × 10^−9^

**Table 2:** The calibration results are displayed in experiment 2.

	Camera 1	Camera 2
**Laser Plane Equation**	$K=\left[\begin{array}{ccc} 3670.82 & 0 & 820.42\\ 0 & 3669.58 & 618.75\\ 0 & 0 & 1 \end{array}\right]$	$K=\left[\begin{array}{ccc} 3682.17 & 0 & 826.36\\ 0 & 3682.67 & 613.59\\ 0 & 0 & 1 \end{array}\right]$
**The Distortion Parameters**	*k*_1_ = −0.11776 *k*_2_ = 0.02398	*k*_1_ = 0.13982 *k*_2_ = 0.02102
**Laser Plane Equation**	−0.560xc + 0.568yc + 0.601zc − 200.46 = 0	−0.523xc − 0.578yc − 0.625zc + 199.52 = 0

**Table 3:** Analysis of calibration accuracy (mm).

		Zhang’s Method		Proposed Method			
				Unrevised		Revised	
**Camera 1**	wi	wm	Δw	wm	Δw	wm	Δw
1	25.00	25.03	0.03	24.97	−0.03	24.98	−0.02
2	50.00	50.05	0.05	49.96	−0.04	49.97	−0.03
3	75.00	50.07	0.07	74.94	−0.06	74.95	−0.05
4	25.00	25.02	0.02	24.97	−0.03	24.98	−0.02
5	50.00	50.05	0.05	49.94	−0.06	49.97	−0.03
6	75.00	75.07	0.07	74.90	−0.10	74.95	−0.05
7	25.00	25.02	0.02	24.97	−0.03	24.98	−0.02
8	50.00	50.04	0.04	49.93	−0.07	49.97	−0.03
9	75.00	75.08	0.08	74.95	−0.05	74.94	−0.06
MAE			0.048		0.052		0.034
RMSE			0.052		0.057		0.037
**Camera 2**							
10	25.00	25.04	0.04	25.03	0.03	25.01	0.01
11	50.00	50.06	0.06	50.04	0.04	50.02	0.02
12	75.00	75.08	0.08	75.05	0.05	75.05	0.05
13	25.00	25.02	0.02	25.03	0.03	25.02	0.02
14	50.00	50.04	0.04	50.05	0.05	50.04	0.04
15	75.00	75.06	0.06	75.07	0.07	75.06	0.06
16	25.00	25.03	0.03	25.05	0.05	25.02	0.02
17	50.00	50.04	0.04	50.08	0.08	50.03	0.03
18	75.00	75.06	0.06	75.11	0.11	75.05	0.05
MAE			0.048		0.057		0.033
RMSE			0.051		0.062		0.037
